# Vasopressin Infusion with Small-Volume Fluid Resuscitation during Hemorrhagic Shock Promotes Hemodynamic Stability and Survival in Swine

**DOI:** 10.1371/journal.pone.0130134

**Published:** 2015-06-24

**Authors:** Raúl J. Gazmuri, Kasen Whitehouse, Karla Whittinghill, Alvin Baetiong, Jeejabai Radhakrishnan

**Affiliations:** 1 Department of Medicine and Resuscitation Institute at Rosalind Franklin University of Medicine and Science, North Chicago, IL, United States of America; 2 Department of Physiology at Rosalind Franklin University of Medicine and Science, North Chicago, IL, United States of America; 3 Critical Care Medicine at Captain James A. Lovell Federal Health Care Center, North Chicago, IL, United States of America; Georgia Regents University, UNITED STATES

## Abstract

**Introduction:**

Current management of hemorrhagic shock (HS) in the battlefield and civilian settings favors small-volume fluid resuscitation before controlling the source of bleeding. We investigated in a swine model of HS the effects of vasopressin infusion along with small-volume fluid resuscitation; with erythropoietin (EPO) and HS severity as additional factors.

**Methods:**

HS was induced in 24 male domestic pigs (36 to 41 kg) by blood withdrawal (BW) through a right atrial cannula modeling spontaneous bleeding by a mono-exponential decay function. The initial 12 pigs received no fluids; the last 12 pigs received normal saline (NS) half the BW volume. Pigs were randomized 2:1 to receive intraosseously vasopressin (0.04 U/kg·min^-1^) or vehicle control from minute 7 to minute 210. Pigs assigned to vasopressin were further randomized 1:1 to receive EPO (1,200 U/kg) or vehicle control and 1:1 to have 65% or 75% BW of their blood volume. Shed blood was reinfused at 210 minutes and the pigs recovered from anesthesia.

**Results:**

Survival at 72 hours was influenced by vasopressin and NS but not by EPO or % BW. Vasopressin with NS promoted the highest survival (8/8) followed by vasopressin without NS (3/8), NS without vasopressin (1/4), and neither treatment (0/4) with overall statistical significance (log-rank test, *p* = 0.009) and each subset different from vasopressin with NS by Holm-Sidak test. Vasopressin increased systemic vascular resistance whereas NS increased cardiac output.

**Conclusion:**

Vasopressin infusion with small-volume fluid resuscitation during severe HS was highly effective enabling critical hemodynamic stabilization and improved 72 hour survival.

## Introduction

Hemorrhagic shock after penetrating trauma in the battlefield accounts for a high percentage of potentially survivable injuries [1]. A report from operations Iraqi Freedom and Enduring Freedom between October 2001 and June 2011 showed that 87% of battlefield fatalities occurred before arrival to a medical treatment facility with 24% deemed potentially survivable [2]. Of these potentially survivable injuries, 91% were associated with hemorrhagic shock.

Fluid resuscitation is hemodynamically effective but it is logistically constrained in the battlefield and not free of complications. In large quantities, fluids can worsen acute traumatic coagulopathy by dilution and hypothermia and dislodge freshly formed clots [3–5]. Accordingly, hemodynamic stabilization with limited fluid resuscitation in victims suffering severe hemorrhagic shock until arrival to a medical treatment facility is considered advantageous and expected to save lives [4,5]. Similar considerations apply to hemorrhage associated with trauma in civilian populations [6,7].

In previous work, we had hypothesized that administration of erythropoietin (EPO) early during hemorrhagic shock could minimize tissue injury through activation of mitochondrial protective mechanisms and help improve resuscitability and survival [8,9]. Although EPO attenuated transient tissue injury it failed to elicit survival benefits [9]. While conducting these studies we observed that vasopressin infusion was highly effective for hemodynamic stabilization and promoted survival under conditions of severe hemorrhagic shock in which aggressive fluid resuscitation had failed. Similar observations have been made by other investigators suggesting that vasopressin could be a highly effective hemodynamic intervention for resuscitation from hemorrhagic shock [10,11]. Our studies further suggested that vasopressin infusion could act in conjunction with small-volume fluid resuscitation to enable sustained hemodynamic stabilization obviating the need to administer large amounts of fluids. The focus of our previous studies, however, was on EPO and the aforementioned observations on the effects of vasopressin were uncontrolled and made in separate series.

We therefore designed a study to specifically investigate the role of vasopressin infusion and small-volume fluid resuscitation for hemodynamic stabilization under conditions of severe hemorrhagic shock maintaining the focus on battlefield relevance and constraints for deployment in far-forward scenarios. The experiments were conducted in a swine model of hemorrhagic shock induced by blood withdrawal through a 14-F cannula advanced into the right atrium. A closed-loop system was developed to model spontaneous bleeding according to a mono-exponential decay function. Shed blood was reinfused at 210 minutes. Resuscitated animals were recovered from anesthesia and observed for up to 72 hours.

We used a factorial design to separately assess the effects of vasopressin infusion, small-volume fluid resuscitation, EPO, and severity of hemorrhagic shock in 24 pigs.

## Materials and Methods

The studies were approved by the Institutional Animal Care and Use Committee (IACUC) at Rosalind Franklin University of Medicine and Science (approval number 12–23) and by the United States Army Medical Research and Materiel Command Animal Care and Use Review Office (ACURO) and were conducted according to institutional guidelines.

### Animal housing and husbandry

Animals were group-housed in the Biological Resource Facility (AAALAC accredited facility) at Rosalind Franklin University of Medicine and Science in which lights are set at the recommended illumination levels with a 12/12 hour cycle controlled via automatic timers. Temperature was maintained between 61 and 81°F. Resting mats and Aspen Sani-Chip bedding from a certified vendor (Harlan Laboratories, Indiana) were used. Assessment for general health and well-being, possible injuries, or death was performed daily by animal care technicians and the day before, during, and after the experiment by the investigators.

### Animal preparation

#### Basic preparation

Twenty-four domestic pigs (36 to 41 kg) were sedated with ketamine hydrochloride (30 mg·kg^-1^ intramuscularly). Anesthesia was induced with propofol (2 mg·kg^-1^ through an ear vein) and the animal intubated with a size 7.5 tracheal tube initiating positive pressure ventilation with a volume controlled ventilator (840 Ventilator System, Nellcor Puritan Bennett, Boulder, CO) set to deliver a tidal volume of 10 ml·kg^-1^ and peak flow of 60 l·min^-1^. Respiratory rate was adjusted to maintain an end-expired PCO_2_ (P_ET_CO_2_) between 35 and 45 mmHg (Capnogard, Novometrix Medical Systems, Wallingford, CT). Anesthesia was continued using isoflurane (1.75% to 2.5%) and a 1:1 mixture of nitrous oxide and oxygen yielding an FiO_2_ of 0.50 adjusted to maintain a surgical plane of anesthesia throughout the experiment. The electrocardiogram was recorded through limb leads using a defibrillator/monitor (Agilent Heartstream XL, Agilent Technologies, Santa Clara, CA). All invasive procedures were performed with sterile technique. A 7-Fr high-fidelity micro-tip catheter transducer (Millar Instruments, Houston, TX) was advanced through the right femoral artery into the descending thoracic aorta for pressure measurement. A balloon-tipped pulmonary artery catheter (Edwards Lifescience Corp, Irvine, CA) was advanced through the left cephalic vein into the pulmonary artery for measuring core temperature and thermodilution cardiac output along with pressures in the right atrium and pulmonary artery. A 6-F high-fidelity micro-tip pressure transducer pigtail catheter (Millar Instruments, Houston, TX) was advanced through the surgically exposed left carotid artery into the left ventricle for pressure measurement. A 14-Fr cannula (Bio-Medicus, Medtronic, Minneapolis, MN) was advanced through the left external jugular vein into the right atrium and used for blood withdrawal. Core temperature was maintained between 37.5°C and 38.5°C with a water-circulated blanket (Blanketrol II, Cincinnati SubZero, Cincinnati, OH). Vasopressin and EPO were administered through the left proximal tibia accessed using an EZ-IO intraosseous infusion system (VidaCare Corp, Santo Antonio, TX).

#### Hemorrhagic shock protocol

Blood was withdrawn into a 2,000 ml blood transfer bag (Charter Medical, Salen, NC) containing heparin (~10 U·ml^-1^ of the anticipated blood volume withdrawn) using a roller pump (model 313S, Watson Marlow, Inc., Wilmington, MA). The heparinized transfer bag was placed on an electronic scale (model 2200, Doran Scales, Inc., Batavia, IL) enabling continuous gravimetric measurement of the rate of blood withdrawal (blood density = 1.06 g/ml). A close-loop system with input from the electronic scale and output to the roller pump was developed by us (RJG and AB) in LabVIEW 6.0. For this application, we used a mono-exponential decay function to mimic spontaneous bleeding after traumatic injury (i.e., high initial bleeding with progressively slower rates as blood pressure declines and hemostatic mechanisms are activated and/or externally applied). The system was highly precise maintaining the target withdrawal volume within 1 ml at any given time during the withdrawal interval. The blood withdrawn was kept in a water bath at 37.5°C and returned over 30 minutes using a blood transfusion filter (PALL Biomedical, Port Washington, NY) at 210 minutes from the start of blood withdrawal to model a situation in which evacuation and arrival to a medical treatment facility from the initial injury is delayed.

#### Recovery from anesthesia and survival

Upon completion of the acute resuscitative phase, all catheters were removed, vessels ligated, and the skin wounds stapled, all under sterile conditions. The animal was allowed to recover from anesthesia and the endotracheal tube removed after resumption of spontaneous breathing. The animal was then returned to its pen and monitored every 60 minutes until it was able to right itself to sternal recumbency. Thereafter the animal was monitored every 4 hours for the initial 24 hours and at a minimum interval of 8 hours until completion of the 72 hours. For analgesia, a fentanyl dermal patch was applied and maintained throughout the 72 hour post-resuscitation period. If additional analgesia was needed, 2.2 mg/kg of flunixin meglumine was administered intramuscularly. The neurological status was evaluated at 24, 48, and 72 hours post-resuscitation using a neurological deficit score (0 = best; 420 = worst) and a cerebral performance category score (1 = normal; 2 = mild disability; 3 = severe disability; and 4 = coma) [12]. The pig was euthanized at 72 hours by intravenous injection of euthanasia solution (pentobarbital sodium and phenytoin sodium; 5 ml, Vedco Inc., St Joseph, MO) or earlier for humanitarian reason in the event of any of the following; moderate to severe pain and distress unalleviated by analgesic agents, inability to eat or drink unassisted after 24 hours post-surgery, non-weight bearing or paralysis after 24 hours, depression or lethargy after 48 hours, profuse diarrhea, infection not resolved with antimicrobial therapy, lack of righting reflex, and cyanosis with difficulty breathing. None of the animals required earlier euthanasia for humane reason.

### Study design

A factorial design was used to separately investigate the effects of vasopressin infusion, small-volume fluid resuscitation, EPO, and the hemorrhagic shock severity. The experiments were randomized by blocks with the investigators blind to the assignments (except for fluid resuscitation), unblinding the assignments only after completion of data collection and verification of data integrity.

**Fig 1:**
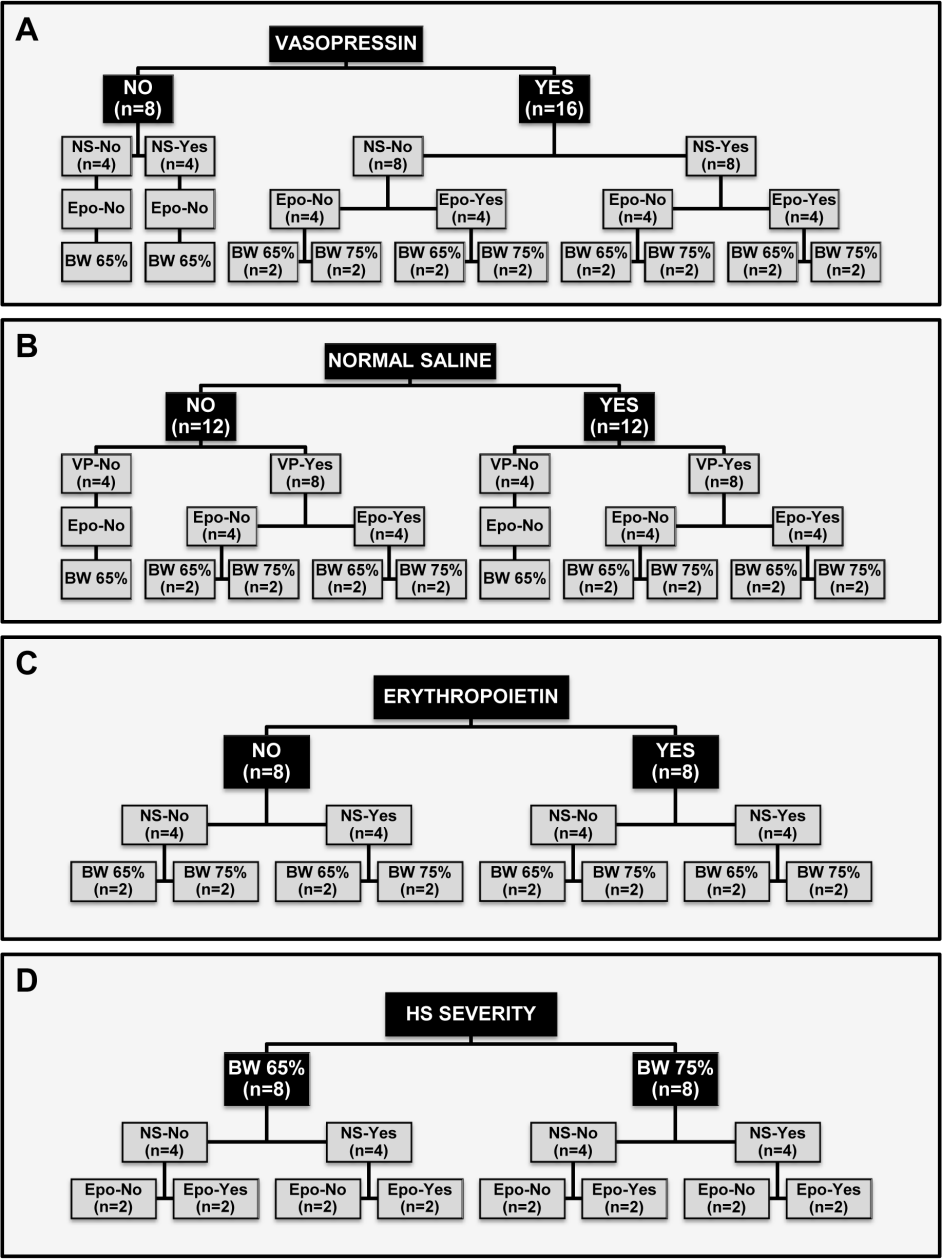
Distributions of the remaining factors for each of the four factor analyses. (**A**) Vasopressin effect, showing the 2:1 randomization with 16 pigs allocated to vasopressin also randomized to normal saline, erythropoietin, and level of hemorrhagic shock severity and 8 pigs allocated to no-vasopressin randomized only to normal saline but not to erythropoietin and exposed to the lowest hemorrhagic shock severity; (**B**) Normal saline effect, showing the 1:1 randomization with 12 pigs allocated to each level and a balanced distribution of the remaining factors; (**C**) Erythropoietin effect for the 16 pigs that received vasopressin, showing the 1:1 randomization with 8 pigs allocated to each level and a balanced distribution of the remaining factors; and (**D**) Hemorrhagic shock severity effect for the 16 pigs that received vasopressin, showing the 1:1 randomization with 8 pigs allocated to each level and a balanced distribution of the remaining factors. NS, normal saline; Epo, erythropoietin; BW, blood withdrawal.

Each of the four factors analyzed and the corresponding distributions of the remaining factors for each of the analyses are shown in Fig 1. The rationale and approach for investigating the aforementioned factors is detailed below.

#### Vasopressin

As discussed above, the addition of vasopressin infusion in an series from a previous study markedly improved initial resuscitation and subsequent 72 hour survival relative to a preceding series in which only fluid resuscitation with normal saline was used before blood reinfusion for the same hemorrhagic shock severity [9]. For the present study, pigs were randomized 2:1 to vasopressin or no vasopressin. Vasopressin (Pitressin, JHP Pharmaceuticals, Rochester, MI) or vehicle control was infused intraosseously using a syringe pump (PHD 2000 Syringe Pump Series, Harvard Apparatus, Holliston, MA) at a constant rate of 0.04 U/kg·min^-1^ from minute 7 of blood withdrawal (i.e., 16% of the blood volume removed) until minute 210 coincident with the start of blood reinfusion.

#### Fluid resuscitation

The preceding study [9] also showed that in the presence of vasopressin initial resuscitability and 72 hour survival could be achieved successfully administering a reduced amount of normal saline (corresponding to half the amount of blood withdrawn) or no fluids at all. Thus, for the present series we also examined whether normal saline could be required. The approach involved starting the series without fluid resuscitation and performing an interim analysis after 12 experiments. The interim analysis showed a 72 hour survival of 25% (3/12) for the initial 12 experiments prompting for the last 12 experiments administration of normal saline from minute 90 to minute 120 through an ear vein. The volume of normal saline administered corresponded to half the volume of blood withdrawn; thus, 19.5 ml/kg was administered when 65% of the blood volume was removed–estimated as 60 ml/kg–and 22.5 ml/kg when 75% of the blood volume was removed.

#### Erythropoietin (EPO)

In keeping with the original objective of our funded project but incorporating knowledge gained from the previous series, in which EPO had no effect on resuscitation and 72 hour survival but attenuated organ dysfunction under conditions of severe but survivable hemorrhagic shock [9], we randomized in the subset of animals receiving vasopressin to also receive EPO or vehicle control. Similar to preceding series, EPO was administered as a single intraosseous bolus dose of 1,200 U/kg after 14% of the blood volume had been removed (i.e., 6 minutes from the start of blood withdrawal).

#### Hemorrhagic shock severity

The severity of hemorrhagic shock was varied in the subset of animals receiving vasopressin by randomizing the blood withdrawal to 65% or to 75% of the blood volume. It is pertinent to highlight that in our previous study the highest amount of blood withdrawn was 65% of the estimated blood volume [9]. A mono-exponential decay function as described above was used for both blood withdrawal targets, stopping after 60 minutes for the 65% and after 80 minutes for the 75% blood withdrawal subsets.

### Measurements

#### Blood analysis

Blood samples were collected from the aorta and pulmonary artery and were processed on site for pH, PO_2_, PCO_2_, hemoglobin, base excess, and lactate using a cartridge based device (OPTI CCA-TS Blood Gas and Electrolyte Analyzer, OPTI Medical Systems, Roswell, GA) and for common hemoglobin types (oxy-, met-, carboxy-, and reduced-) using a co-oximeter (AVOXimeter 4000, AVOX systems Inc., San Antonio, TX). O_2_ content in the aorta (CaO_2_) and pulmonary artery (CvO_2_) was calculated according to the following equation:

$$O_{2}\;Content\;(\frac{ml}{dl})=Hemoglobin\;(\frac{g}{dl})\times 1.39\;(\frac{ml}{g})\times S_{F}O_{2}+0.003\;(\frac{ml}{dl}\cdot {mmHg}^{-1})\times PO_{2}\;(mmHg)$$

where 1.39 denotes ml of O_2_ bound to 1 g of hemoglobin (Hufner’s number), S_F_O_2_ the fraction of oxyhemoglobin relative to the four hemoglobin types, and 0.003 the O_2_ solubility coefficient. Oxygen delivery and consumption index were calculated from CaO_2_ multiplied by cardiac index and from the difference between CaO_2_ and CvO_2_ multiplied by cardiac index respectively, both estimated in ml/min·m^-2^. Additional blood from the aortic samples had their plasma separated and stored at -80°C for subsequent batch analysis of glucose, urea nitrogen, creatinine, alanine aminotransferase, aspartate aminotransferase, alkaline phosphatase, creatine kinase, and cardiac troponin I at the Captain James A. Lovell Federal Health Care Center, North Chicago, IL.

#### Hemodynamic measurements

Thermodilution cardiac output was measured in duplicate after bolus injection of 0.9% NaCl (5 ml) into the right atrium (HP-Philips M012AT cardiac output module, Amsterdam, The Netherlands). Cardiac output was normalized to body surface area using the Kelley equation (body surface area [m^2^] = 0.073·body-weight^2/3^ [kg]) [13]. Aortic and left ventricular pressure signals were calibrated and zeroed using a Millar PCU 2000 Box. Pressure signals measured using fluid-filled systems were calibrated using a digital pressure gauge (DPG1000, Omega Engineering) and zeroed to mid-cavity level. All signals were sampled and digitized at 250 Hz using a 16-bit data acquisition board (AT-MIO-16XE-50; National Instruments, Austin, TX) and analyzed using custom-developed software (Labview 6.0, National Instruments).

#### Cardiac function

Indices of cardiac function were derived from left ventricular and pulmonary artery pressures, reporting the left and right ventricular stroke work index (LVSWI and RVSWI, respectively) corresponding to the stroke volume index times the difference between systolic and end-diastolic left ventricular pressures and between the mean pulmonary and right atrial pressures, respectively, expressed in centijoules (cJ) by multiplying by 0.013332 [14]. The systemic vascular resistance index (SVRI) was calculated from the difference between mean aortic and mean right atrial pressure divided by cardiac index and reported in dynes·s/cm^-5^·m^-2^.

### Statistical analysis

The statistical analysis was performed using Sigmaplot 11.0 (Systat Software, Inc., San Jose, CA). The survival effect of each of the four studied factors was analyzed using the Kaplan-Meier method and the log-rank test using the Holm-Sidak pairwise multiple comparisons test when applicable. The risk contribution of each of the studied factors on survival time was analyzed using the Cox proportional hazard analysis. Because only vasopressin and normal saline had an independent survival effect, the analysis of explanatory (hemodynamic and metabolic) variables was limited to these two factors using two-way repeated ANOVA analyzing the separate effects of vasopressin and normal saline identifying main effects, interactions, and differences at specific time points. Samples sizes for the separate factor analyses of 8 or higher per group were deemed adequate to identify clinically relevant effects based in previous work with similar experimental protocols. Data are shown as means ± SEM in figures and ± SD in tables and text. A two-tail *p* ≤ 0.05 was considered statistically significant.

## Results

No unexpected adverse events occurred. Demise was the consequence of hemorrhagic shock severity during the acute phase. Animals that survived the acute phase and were recovered from anesthesia survived the 72 hour observation interval recovering baseline neurological function and overall performance.

### Baseline

No significant differences among groups were observed at baseline (Tables 1 and 2) except for cardiac index and indices of left and right ventricular work that were higher in the group assigned to receive normal saline.

**Table 1:** Effect of vasopressin on indices of organ function/injury.

	Baseline	BW	HS	End BR	Survival
	-10 min	60 min	150 min	240 min	72 h
**Glucose (mg/dl)**					
No-Vasopressin	95±21^[^ ^8^ ^]^	119±70^[^ ^8^ ^]^	185±34^[^ ^3^ ^]^	143±7^[^ ^3^ ^]^	118^[^ ^1^ ^]^
Vasopressin	103±54^[^ ^16^ ^]^	228±140^[^ ^16^ ^]^ ^†^ ^b^	137±102^[^ ^12^ ^]^	92±59^[^ ^11^ ^]^	107±7^[^ ^11^ ^]^
**Blood Urea Nitrogen (mg/dl)**					
No-Vasopressin	11±3	12±3	16±3^a^	16±3^a^	9
Vasopressin	8±3	11±3^b^	15±4^b^	14±4^b^	8±2
**Creatinine (mg/dl)**					
No-Vasopressin	0.9±0.1	1.3±0.1^b^	1.9±0.4^b^	1.9±0.4^b^	0.8
Vasopressin	1.0±0.1	1.4±0.2^b^	1.8±0.5^b^	1.7±0.4^b^	0.9±0.1
**Aspartate Aminotransferase (U/l)**					
No-Vasopressin	43±11	43±11	48±3	62±4	54
Vasopressin	38±9	32±12	77±144	93±112	140±117
**Alanine Aminotransferase (U/l)**					
No-Vasopressin	69±14	65±14	62±5	72±8	104
Vasopressin	64±8	58±12	50±6	60±8	178±74* ^b^
**Alkaline Phosphatase (U/l)**					
No-Vasopressin	196±38	193±36	236±47	245±47	200
Vasopressin	199±60	193±53	236±70^a^	254±85^b^	200±66
**Creatine Kinase (U/l)**					
No-Vasopressin	1325±923	1384±817	1458±438	1277±192	1479
Vasopressin	1352±489	1337±432	1456±608	1574±675	8443±7679
**Cardiac Troponin I (ng/dl)**					
No-Vasopressin	0.04±0.05	0.10±0.11	1.32±0.59^b^	1.43±0.80^b^	0.04
Vasopressin	0.03±0.05	0.03±0.05	0.11±0.18^‡^	0.10±0.16^‡^	0.22±0.33

Numbers in brackets indicate the sample size reflecting animals alive at the specific time point except for HS 150 minute in the no-vasopressin group in which two samples were not available. Values are mean SD. BW, blood withdrawal; HS, hemorrhagic shock; BR, blood reinfusion. The data was analyzed using two-way repeated measures ANOVA reporting statistically significant differences between groups at specified time points

*p≤0.05

^†^ *p≤*0.01, and

^‡^ *p≤*0.001, and statistically significant differences from baseline within each group using the Holm-Sidak test for multiple comparisons

^a^ *p≤*0.01, and

^b^ *p≤*0.001.

**Table 2:** Effect of normal saline on indices of organ function/injury.

	Baseline	BW	HS	End BR	Survival
	-10 min	60 min	150 min	240 min	72 h
**Glucose (mg/dl)**					
No-Normal Saline	104±60^[^ ^12^ ^]^	202±141^[^ ^12^ ^]^ ^b^	172±97^[^ ^5^ ^]^	112±49^[^ ^4^ ^]^	104±8^[^ ^3^ ^]^
Normal Saline	99±25^[^ ^12^ ^]^	181±126^[^ ^12^ ^]^ ^a^	134±94^[^ ^10^ ^]^	100±62^[^ ^10^ ^]^	109±8^[^ ^9^ ^]^
**Blood Urea Nitrogen (mg/dl)**					
No-Normal Saline	10±2	12±3^b^	18±3^c^	17±3^c^	9±3
Normal Saline	8±3	10±4^b^	13±4^c^	14±4^c^	8±2
**Creatinine (mg/dl)**					
No-Normal Saline	1.0±0.1	1.3±0.1^c^	2.0±0.5^c^	1.7±0.3^c^	0.9±0.2
Normal Saline	1.0±0.1	1.4±0.2^c^	1.7±0.4^c^	1.7±0.4^c^	0.9±0.1
**Aspartate Aminotransferase (U/l)**					
No-Normal Saline	36±10	31±14	36±19	53±35	106±67
Normal Saline	43±8	40±10	89±157	100±115	141±128^b^
**Alanine Aminotransferase (U/l)**					
No-Normal Saline	64±12	59±16	50±10	58±15	153±56^c^
Normal Saline	68±8	61±9	54±6	64±6	178±81^c^
**Alkaline Phosphatase (U/l)**					
No-Normal Saline	185±46	180±43	194±58	206±61	228±45^a^
Normal Saline	211±58	206±50	257±60^b^	271±77^c^	191±68^a^
**Creatine Kinase (U/l)**					
No-Normal Saline	1186±444	1193±335	1439±468	1384±439	9541±7322^c^
Normal Saline	1501±791	1512±716	1465±629	1593±711	7304±8028^c^
**Cardiac Troponin I (ng/dl)**					
No-Normal Saline	0.04±0.05	0.05±0.08	0.32±0.34^a^	0.29±0.35^a^	0.05±0.03
Normal Saline	0.02±0.05	0.05±0.09	0.37±0.67	0.43±0.76^a^	0.24±0.35

Numbers in brackets indicate the sample size reflecting animals alive at the specific time point except for HS 150 minute in the no-normal saline group in which two samples were not available. Values are mean SD. BW, blood withdrawal; HS, hemorrhagic shock; BR, blood reinfusion. The data was analyzed using two-way repeated measures ANOVA reporting statistically significant differences from baseline within each group using the Holm-Sidak test for multiple comparisons

^a^ *p≤*0.05

^b^ *p≤*0.01, and

^c^ *p≤*0.001.

There were no statistically significant differences between groups at any specified time point.

### Survival

**Fig 2:**
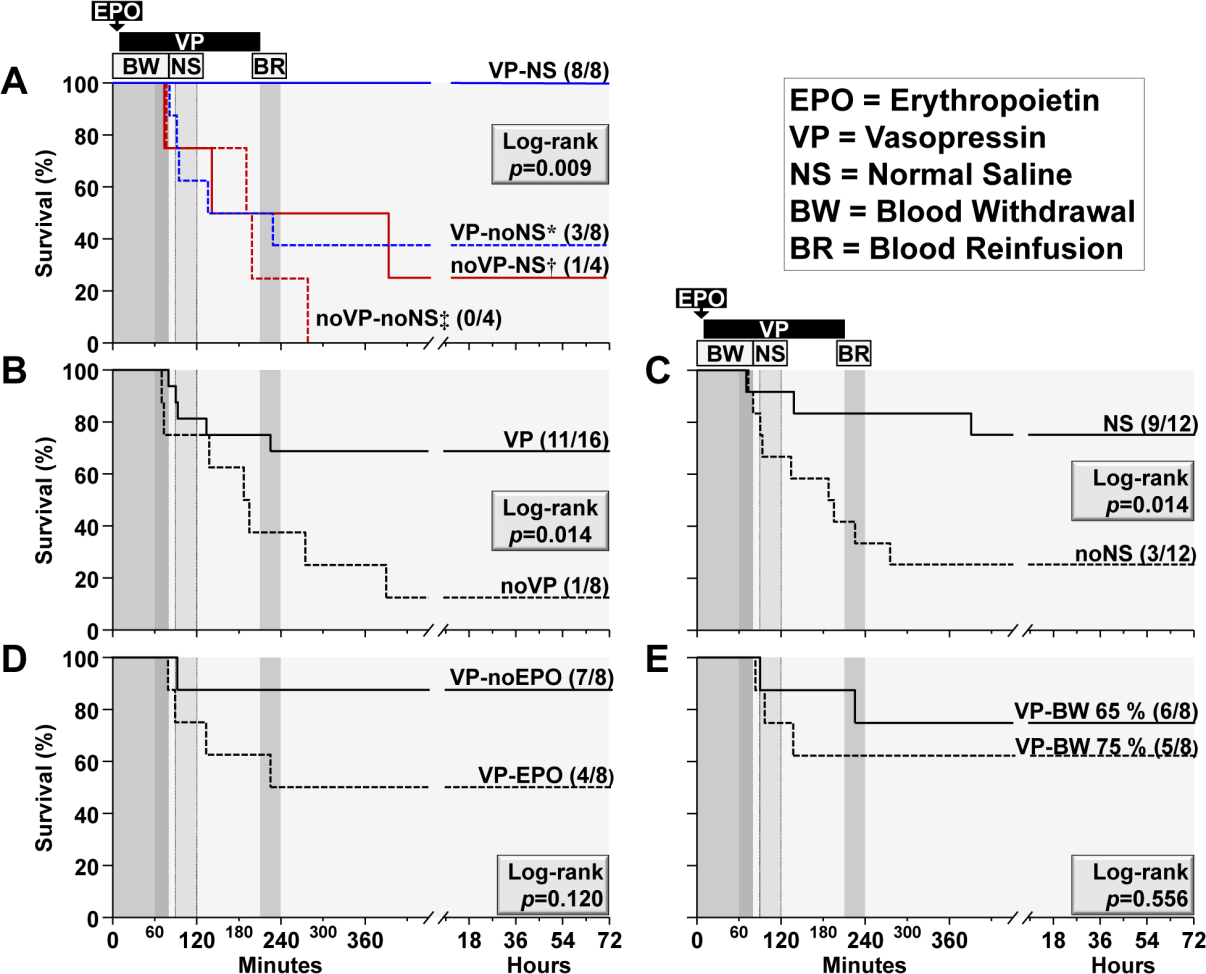
Survival analysis. Kaplan-Meier survival curves analyzed using the log-rank test and the Holm-Sidak test for multiple comparisons when applicable. The *p*-value for each log-rank test results is shown inside each graph. (**A**) Kaplan-Maier survival curves comparing the effects of vasopressin with normal saline (VP-NS), VP without NS (VP-noVP), no VP with NS (noVP-NS), and neither VP nor NS (noVP-noNS). Pairwise comparisons by the Holm-Sidak test demonstrated significantly lower survival for VP-noNS (*p = 0.035), noVP-NS (**†** *p* = 0.0198), and noVP-noNS (**‡** *p* = 0.001) compared with VP-NS. B through E show Kaplan-Maier survival curves for the independent effects of vasopressin (**B**), normal saline (**C**), erythropoietin (**D**), and hemorrhagic shock severity (**E**) with the remaining factors allocated as shown in Fig 1. In parentheses, number of animals alive at 72 hour survival relative to the initial subset.

Fig 2 shows the survival effect of vasopressin and normal saline, demonstrating a significantly higher survival rate associated with the combination of vasopressin and normal saline compared to each of the individual interventions or to none of them (Fig 2A). In addition, separate survival analyses performed for each of the main factors while maintaining a balanced distribution of the remaining factors showed a survival benefit associated with vasopressin infusion (Fig 2B) and normal saline (Fig 2C). Use of EPO (Fig 2D) and increasing hemorrhagic shock severity (Fig 2E) had no statistically significant impact on survival.

### Proportional hazard risk

Table 3 shows hazard ratios for each factor demonstrating a significantly reduced risk on survival time associated with administration of vasopressin and administration of normal saline but not EPO or hemorrhagic shock severity.

**Table 3:** Hazard ratios for main factors.

Covariate	Coefficient	SE	*p*-value	Hazard Ratio	95%CI
Vasopressin	-3.02	1.24	0.015	0.049	0.004–0.559
Normal Saline	-1.88	0.77	0.014	0.152	0.034–0.688
Erythropoietin	2.20	1.17	0.060	8.989	0.910–88.766
Blood Withdrawal 75%	0.84	0.93	0.365	2.324	0.374–14.435

### Effects of vasopressin on hemodynamic and metabolic parameters

**Fig 3:**
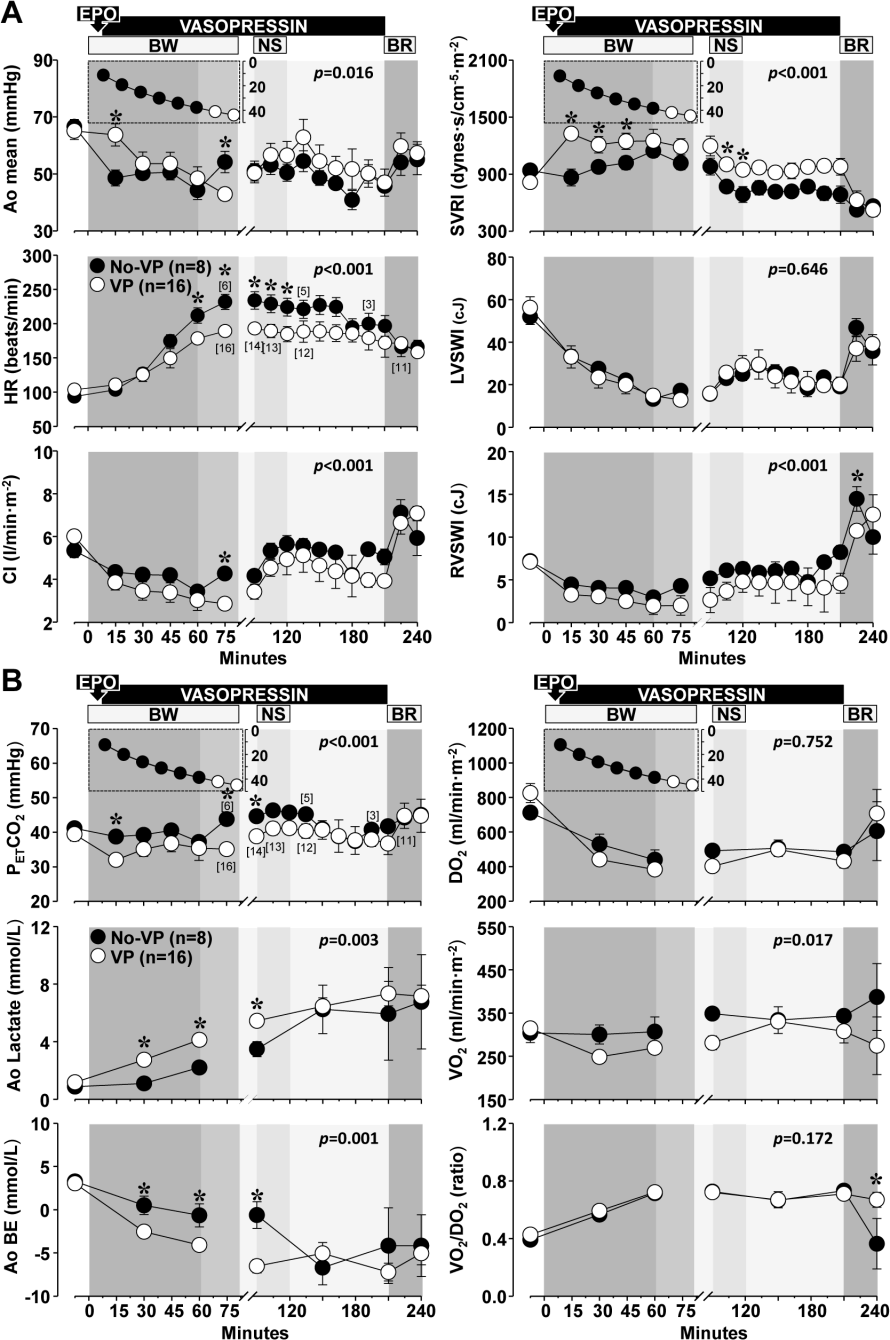
Effects of vasopressin on hemodynamic and metabolic function during hemorrhagic shock. EPO, erythropoietin; BW, blood withdrawal; NS, normal saline; BR, blood reinfusion. The inset depicts the time course of the blood withdrawal (ml/kg) ending at 60 minutes in the 65% BW subset and at 80 minutes in the 75% BW group. Numbers in brackets indicate when the number of animals decreased from the preceding time point. Values are shown as mean ± SEM. Differences between groups were analyzed by two-way repeated measures ANOVA. Overall statistical significances for the treatment effect are shown inside each graph. *p ≤ 0.05 denotes statistically significant differences at the specified time point. Open circles denote vasopressin (VP) and closed circles denote vehicle control (No-VP). (**A**) Effects on hemodynamic and myocardial function. Ao, aortic pressure; HR, heart rate; CI, cardiac index; SVRI, systemic vascular resistance index; LVSWI, left ventricular stroke work index; RVWI, right ventricular stroke work index. (**B**) Effects on metabolic variables. P_ET_CO_2_, end-tidal carbon dioxide; Ao, aortic; BE, base excess; DO_2_, oxygen delivery index; VO_2_, oxygen consumption index; VO_2_/DO_2_, oxygen consumption and delivery ratio. There was an overall statistically significant interaction between treatment and time for Ao BE (*p* = 0.040).

Fig 3 depicts the hemodynamic and metabolic effects of vasopressin. Vasopressin increased systemic vascular resistance resulting in an initial increase in mean aortic pressure attaining statistical significance at minute 15 from the start of blood withdrawal. However, vasopressin also blunted the chronotropic response to hemorrhagic shock attaining statistical significance from minute 60 to minute 120, further reducing cardiac index despite no adverse effect on left ventricular work index. This effect precluded a sustained increase in mean aortic pressure (Fig 3A). Vasopressin intensified lactic acidosis and the base excess deficit, likely related to a flow dependent reduction in systemic oxygen consumption (VO_2_), but this effect was transient attaining values similar to pigs treated without vasopressin before blood reinfusion (Fig 3B). The P_ET_CO_2_ largely followed changes in cardiac index consistent with its flow dependency under low-flow states (Fig 3B). Blood reinfusion restored cardiac index to baseline halting the progression of lactic acidosis initiating the reversal of hemodynamic and metabolic abnormalities.

### Effects of vasopressin on organ function/injury

These changes are shown on Table 1. Blood glucose increased during hemorrhagic shock attaining statistical significance only in vasopressin-treated pigs normalizing by 72 hours. Blood urea nitrogen and creatinine increased mildly in both groups during hemorrhagic shock normalizing by 72 hours. A statistically insignificant increase in aspartate aminotransferase was observed associated with increases in alkaline phosphatase during hemorrhagic shock followed by an increase in alanine aminotransferase by 72 hours in vasopressin treated pigs. There was also a statistically insignificant increase in creatine kinase at 72 hours in vasopressin treated pigs. There was a mild increase in cardiac troponin I during hemorrhagic shock in control pigs but not in vasopressin-treated pigs.

### Hemodynamic and metabolic effects of normal saline

**Fig 4:**
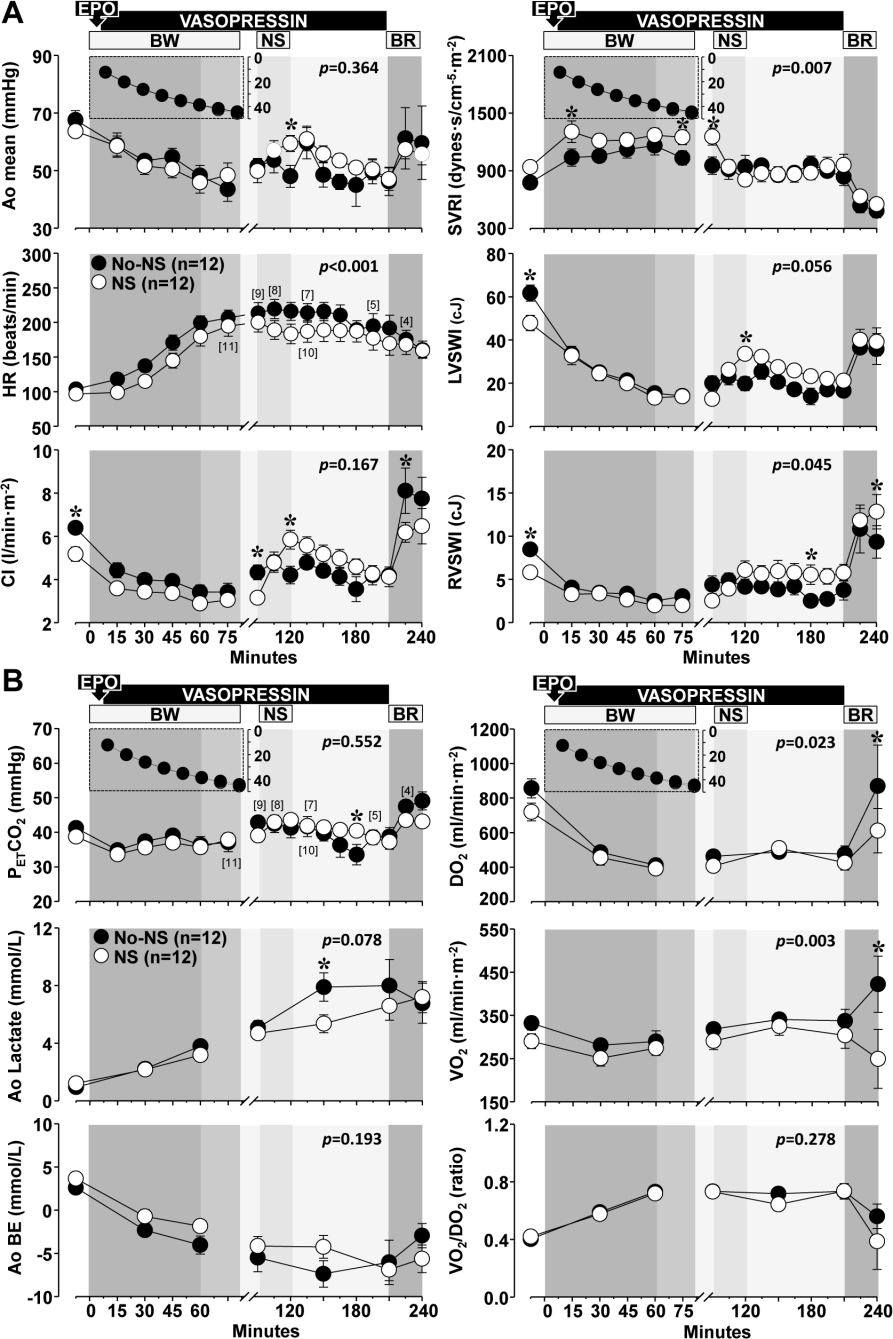
Effects of normal saline on hemodynamic and metabolic function during hemorrhagic shock. EPO, erythropoietin; BW, blood withdrawal; NS, normal saline; BR, blood reinfusion. The inset depicts the time course of the blood withdrawal (ml/kg) ending at 60 minutes in the 65% BW subset and at 80 minutes in the 75% BW group. Numbers in brackets indicate when the number of animals decreased from the preceding time point. Values are shown as mean ± SEM. Differences between groups were analyzed by two-way repeated measures ANOVA. Overall statistical significances for the treatment effect are shown inside each graph. *p ≤ 0.05 denotes statistically significant differences at the specified time point. Open circles denote normal saline (NS) and closed circles denote no fluid administration (No-NS). (**A**) Effects on hemodynamic and myocardial function. Ao, aortic pressure; HR, heart rate; CI, cardiac index; SVRI, systemic vascular resistance index; LVSWI, left ventricular stroke work index; RVWI, right ventricular stroke work index. There was an overall statistically significant interaction between treatment and time for CI (*p* < 0.001), LVSWI (*p* = 0.007), and RVWI (*p* = 0.009). (**B**) Effects on metabolic variables. P_ET_CO_2_, end-tidal carbon dioxide; Ao, aortic; BE, base excess; DO_2_, oxygen delivery index; VO_2_, oxygen consumption index; VO_2_/DO_2_, oxygen consumption and delivery ratio.

Fig 4 depicts the hemodynamic and metabolic effects of normal saline. There were group differences before administration of normal saline likely reflecting the sequential allocation to treatment with normal saline in the last half of the series. Coincident with administration of normal saline from minute 90 to 120, left ventricular work index increased–attributed to preload augmentation–leading to a higher cardiac index and lowering the systemic vascular resistance index (Fig 4A). This effect was associated with attenuation of lactic acidosis (Fig 4B). Upon return of shed blood, animals treated with normal saline had hemodynamic and metabolic parameters closer to baseline.

### Effects of normal saline on organ function/injury

These changes are summarized on Table 2 and were similar to those described above in relation to the vasopressin effects; namely, increases in glucose, blood urea nitrogen, creatinine, and cardiac troponin I during hemorrhagic shock that reversed by 72 hours but without differences between groups. Aspartate aminotransferases increased at 72 hours in normal saline treated pigs. Alanine aminotransferase increased in both groups also by 72 hours preceded by increases in alkaline phosphatase. Elevation in creatine kinase occurred in both groups by 72 hours.

### Neurological deficit and overall performance

Pigs that survived 72 hours exhibited minimal or no clinical neurological deficits and had adequate overall performance.

### Effect of EPO

Within the subset of pigs that received vasopressin, EPO failed to confirm a previously reported beneficial effect attenuating acute organ injury. In fact, pigs that received EPO had a lower mean aortic pressure, a blunted inotropic response, a higher systemic oxygen extraction ratio, and higher levels of aspartate aminotransferase and alkaline phosphatase during hemorrhagic shock. Their neurological deficit score was higher and overall performance category worse at 24 hours returning to baseline by 72 hours. Although not statistically significant, survival was lower in pigs that received EPO.

## Discussion

The present study demonstrates a marked survival benefit associated with early and sustained administration of vasopressin in conjunction with small-volume fluid resuscitation in a swine model of severe hemorrhagic shock. Vasopressin acted by increasing systemic vascular resistance whereas normal saline acted by increasing cardiac index through a preload effect. Although vasopressin intensified lactic acidosis, this effect was counterbalanced by normal saline. The combination of vasopressin and normal saline averted early demise and secured sufficient hemodynamic stability for the 210 minutes of hemorrhagic shock before blood reinfusion, modeled to simulate delayed arrival to a medical treatment facility where control of the bleeding source and resuscitation with blood products could be performed. Administration of EPO conferred no additional survival benefit.

### Vasopressin

Vasopressin is a nonapeptide synthesized by the paraventricular and supraoptic nuclei in the hypothalamus and stored in the posterior pituitary. It is released in response to osmotic and baroreceptor signaling. In the circulation, vasopressin acting *via* V_1_ receptors on smooth muscle cells increases vascular tone and promotes vasoconstriction. The effect is mediated in part through closure of ATP sensitive K^+^ channels preventing cell membrane hyperpolarization and thus maintaining calcium channels operational, making cytosolic calcium available for contraction. Vasopressin also blunts increases in cyclic guanosine monophosphate and reduces NO synthase production [11,15]. The vascular effects are predominantly arteriolar–increasing vascular resistance with relative minor effects on venous tone [16]. Vasopressin is released during hemorrhagic shock and other hemodynamic crises as part of the neuroendocrine stress response; however, the reserve of vasopressin is limited and exogenous administration has been suggested for hemodynamic stabilization in sepsis [17–19] and hemorrhagic shock [11,20,21]. In previous studies also using swine models of hemorrhagic shock, vasopressin was comparably superior to fluid administration and to epinephrine [10,22]. In a dog model of hemorrhagic shock, vasopressin given before administering fluids was more effective that when used after fluid administration [23]. In our own previous studies using a similar swine model of controlled hemorrhagic shock, we observed a marked survival effect produced by vasopressin infusion under conditions in which aggressive fluid resuscitation failed [9]. Vasopressin has indeed been proposed as a “preferred” vasopressor agent for hemodynamic stabilization during hemorrhagic shock [11].

The dose chosen for the present studies followed studies by Voelckel *et al*., also in swine [10]. We began the vasopressin infusion at 7 minutes into hemorrhagic shock based on previous experience with the model in which demise started after removing more than 50% of the blood volume using a linear function. The mono-exponential decay function chosen to model spontaneous bleeding resulted in faster earlier blood loss. Thus, we elected to start the vasopressin infusion before critical reduction in blood volume occurred. We assumed that such approach would be deployable in the battlefield through the intraosseous route using a small pre-loaded and pre-programmed infusion pump upon recognition of severe bleeding with the option of administering fluids as a secondary intervention guided in the battlefield by combat casualty care criteria.

Consistent with previous observations [24], vasopressin blunted the baroreceptor response to hypovolemia such that not only the heart rate but also the cardiac index was lower than in vehicle control animals (Fig 3A). This effect was associated with a higher oxygen extraction ratio and higher lactate levels early during hemorrhagic shock but without compromising the ability of vasopressin to promote sustained hemodynamic stability and improve survival without organ injury or dysfunction. Moreover, vasopressin infusion appeared to have protected the myocardium as minor increases in cardiac troponin I were observed only in control animals (Table 1).

### Fluid resuscitation

The aggressiveness of fluid administration during hemorrhagic shock before control of the bleeding source is been tempered in both military [4,5] and civilian settings [6,7]. Aggressive fluid administration can compromise coagulation by dilution of clotting factors and hypothermia. Concomitantly, there is activation of the fibrinolytic system, tipping the hemostatic balance toward bleeding and prompting greater use of blood products [25]. Excessive fluid administration also risks pulmonary edema and edema in other territories contributing to the development of compartment syndromes and intestinal dysfunction [26]. There is also concern that when normal saline is the fluid administered in large amounts it can precipitate renal injury through development of hyperchloremic acidosis [27] and also prompt activation of inflammatory cascades [28]. In addition, it is logistically disadvantageous to carry large amounts of fluids for deployment in the battlefield. For these reasons, small-volume fluid resuscitation is gaining acceptance in military and civilian settings with clinical trials showing better outcomes compared to aggressive fluid resuscitation protocols [6,7].

The present study showed that small-volume fluid resuscitation could be highly effective when used in conjunction with vasopressin infusion during severe hemorrhagic shock. In the present study each of the 8 pigs treated with vasopressin infusion and small-volume fluid resuscitation survived 72 hours without evidence of organ dysfunction.

### Limitations

The main limitation of the present study is the absence of tissue injury that typically accompanies hemorrhagic shock in the battlefield and the ensuing inflammatory response. In addition, the necessity of anesthesia could mask some of the physiologic responses to hypovolemic shock. Finally, the naturally occurring neuropeptide in swine is lysine vasopressin whereas in humans it is arginine vasopressin. Thus, potency of the effect may vary contingent on specific receptors and vascular beds and translation of these findings to humans will need to consider human dose-responses.

## Conclusions

The present findings support the concept that early and sustained administration of vasopressin could be highly effective for hemodynamic stabilization in the setting of severe hemorrhagic shock and work in conjunction with small-volume fluid resuscitation to initiate and maintain critical hemodynamic stability until arrival to a medical treatment facility.

## Supporting Information

S1 Dataset The primary dataset pertaining to this study. (XLSX)
